# Physical Biology of Axonal Damage

**DOI:** 10.3389/fncel.2018.00144

**Published:** 2018-06-06

**Authors:** Rijk de Rooij, Ellen Kuhl

**Affiliations:** Department of Mechanical Engineering and Bioengineering, Stanford University, Stanford, CA, United States

**Keywords:** tau protein, microtubules, slip bonds, diffuse axonal injury, neurodegeneration, multiscale modeling, finite element analysis

## Abstract

Excessive physical impacts to the head have direct implications on the structural integrity at the axonal level. Increasing evidence suggests that tau, an intrinsically disordered protein that stabilizes axonal microtubules, plays a critical role in the physical biology of axonal injury. However, the precise mechanisms of axonal damage remain incompletely understood. Here we propose a biophysical model of the axon to correlate the dynamic behavior of individual tau proteins under external physical forces to the evolution of axonal damage. To propagate damage across the scales, we adopt a consistent three-step strategy: First, we characterize the axonal response to external stretches and stretch rates for varying tau crosslink bond strengths using a discrete axonal damage model. Then, for each combination of stretch rates and bond strengths, we average the axonal force-stretch response of *n* = 10 discrete simulations, from which we derive and calibrate a homogenized constitutive model. Finally, we embed this homogenized model into a continuum axonal damage model of [1-d]-type in which d is a scalar damage parameter that is driven by the axonal stretch and stretch rate. We demonstrate that axonal damage emerges naturally from the interplay of physical forces and biological crosslinking. Our study reveals an emergent feature of the crosslink dynamics: With increasing loading rate, the axonal failure stretch increases, but axonal damage evolves earlier in time. For a wide range of physical stretch rates, from 0.1 to 10 /s, and biological bond strengths, from 1 to 100 pN, our model predicts a relatively narrow window of critical damage stretch thresholds, from 1.01 to 1.30, which agrees well with experimental observations. Our biophysical damage model can help explain the development and progression of axonal damage across the scales and will provide useful guidelines to identify critical damage level thresholds in response to excessive physical forces.

## 1. Introduction

Brain injury is a major cause of disability and death that is often triggered by an external impact to the head (Hyder et al., 2007; Taylor, 2017). This impact can consist of a single, severe event that immediately leads to traumatic brain injury, or of repeated mild events that gradually result in chronic traumatic encephalopathy. In both cases, the effect of the impact manifests itself at a much smaller scale in the brain: *the scale of the axon* (Johnson et al., 2013; Smith and Meaney, 2016).

**Figure 1:**
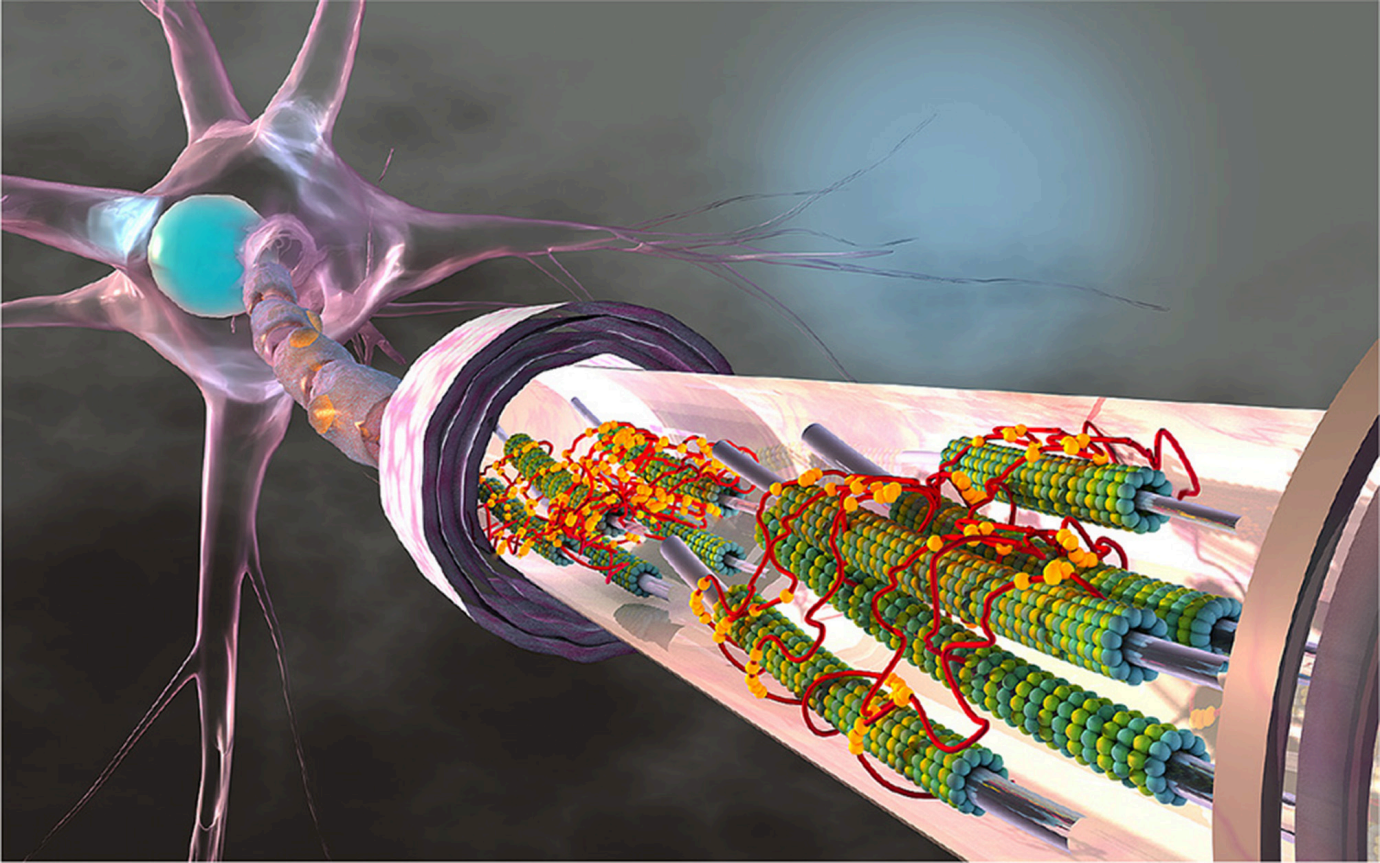
The axon is a long and slender protrusion from the neuronal cell body that consists of a system of longitudinally aligned microtubules. Microtubules are composed of heterodimers of α- and β-tubulin, shown in green and blue, that form 13 laterally joined protofilaments, each up to 100 μm long. Axons can extend several centimeters in length and their microtubules never run continuously from the cell body to the distal end. Instead, they form overlapping segments with 10–50 microtubules in any given cross section. Neuronal microtubules are stabilized and cross-linked by tau proteins, shown in red, which bind to microtubules with their three or four binding repeats, shown in yellow. Several components of the axon including neurofilaments, other crosslinking proteins, and cytoskeletal organelles are not displayed.

The axon is part of the nerve cell, the neuron, that further consists of a cell body with the cell nucleus and synapses that form connections with other neurons. Figure 1 illustrates a typical axon as a long and slender protrusion from the cell body to provide signaling pathways and transport highways within and away from the brain (Debanne et al., 2011). The axonal cytoskeleton consists of a system of longitudinally aligned microtubules and neurofilaments (Ouyang et al., 2013; Kirkcaldie and Collins, 2016) surrounded by an actin cortex and layers of fatty material, the myelin sheath. Axonal microtubules are composed of heterodimers of α- and β-tubulin that form 13 laterally joined protofilaments, each up to 100μm long (Alberts et al., 2014). These microtubules never run continuously from the cell body to the distal end of the axon. Instead, they form overlapping segments with 10–50 microtubules in any given cross section (Krieg et al., 2017). Microtubules are interconnected by active and passive crosslinking proteins including dynein, kinesin, and tau (Coles and Bradke, 2015). Recent studies suggest that tau protein plays a major role in various types of neurodegeneration that are collectively recognized as *tauopathies*. Classical examples include Alzheimer's disease, Pick's disease, progressive supranuclear palsy, and chronic traumatic encephalopathy (Woerman et al., 2016; Eisenberg and Sawaya, 2017). A classical hallmark of chronic traumatic encephalopathy is an abnormal increase of tangled tau protein across the brain (Mez et al., 2017). Yet, the precise cause, development, and diagnosis of chronic traumatic encephalopathy are only incompletely understood and remain active fields of research (Asken et al., 2017).

Physical forces play an important role in the axon under physiological conditions (Suter and Miller, 2011; O'Toole et al., 2015). However, beyond a critical level, forces can trigger axonal degradation and damage (van den Bedem and Kuhl, 2015). Indeed, physical impacts to the head that result in excessive axonal stretch (Ji et al., 2014) may trigger a gradual degradation of the tau-microtubule complex (van den Bedem and Kuhl, 2017). Tau protein is an intrinsically disordered protein with three or four binding repeats that bind to neuronal microtubules and prevents them from depolymerization (Kadavath et al., 2015). Bound tau protein is believed to form an electrostatic zipper with tau protein from neighboring microtubules (Fitzpatrick et al., 2017). As such, tau plays a critical role in stabilizing individual microtubules (Chung et al., 2015) and forming microtubule bundles (Choi et al., 2016). Increasing evidence suggests that axonal damage develops when a physical force is large enough to break the tau-microtubule bonds. An excessive loss of tau crosslinks results in the depolymerization of microtubules (Kadavath et al., 2015), the disintegration of microtubule bundles (Krieg et al., 2017), and the disruption of axonal transport (Tang-Schomer et al., 2012). While experimental testing of the structural integrity of the tau-microtubule complex remains challenging (Li et al., 2015), computational modeling of the axon in response to physical forces can provide useful mechanistic insight into these causal relations (de Rooij and Kuhl, 2018). Although all cytoskeletal elements contribute to the mechanical properties of the axon (Kirkcaldie and Collins, 2016), recent studies suggest that the mechanical stiffness of the axon is most reduced when disrupting axonal microtubules, followed by neurofilaments and microfilaments (Ouyang et al., 2013). Mechanical models of the axon have therefore mainly focused on microtubules (Suter and Miller, 2011), which, because of their hollow circular cross section, provide the largest resistance to bending and tension (Howard, 2001). Models of the axon exist at various levels of complexity ranging from a combination of rheological spring and dashpot elements (O'Toole et al., 2008) via a discrete arrangement of microtubules and crosslinks (Peter and Mofrad, 2012; Ahmadzadeh et al., 2015; Jakobs et al., 2015; Lazarus et al., 2015) to a continuum representation of the axon as a whole (Recho et al., 2016; García-Grajales et al., 2017). Our group has recently proposed a new axonal damage model that integrates the dynamics of microtubule polymerization and depolymerization, the biology of crosslink attachment and detachment, and physics of stretching using a custom-designed finite element model (de Rooij et al., 2016; de Rooij and Kuhl, 2018).

Although brain damage has its mechanistic origin at the axon level, the severity of a head impact is often quantified at the whole brain level by applying experimentally measured linear and rotational accelerations to a human head model (Kuo et al., 2017). In these models, it is essential to accurately capture the brain geometry (Kleiven and von Holst, 2002; Takhounts et al., 2003) and its material properties (de Rooij and Kuhl, 2016; Budday et al., 2017). The most critical input to these models, however, is the critical strain or stretch level beyond which axonal damage occurs (Bain et al., 2004). To better understand the propagation of axonal damage across the scales, we have to connect the axon level to the whole brain level. Toward this goal, we simulate the effect of physical forces across the axon and derive a continuum model for axonal damage as function of the applied stretch and stretch rate. Central to our model is the classical Bell model (Bell, 1978) that characterizes the dynamics of the tau-microtubule complex, from which we infer a damage evolution law that can be easily embedded into finite element models at the whole brain level. Our work provides a systematic strategy to mechanistically correlate crosslink dynamics on the microscopic scale to the evolution of axonal damage on the mesoscopic scale. We anticipate that this work will provide insight into the development of brain damage across the scales and improve current modeling techniques to quantify brain damage for a given physical impact to the brain.

## 2. Methods

### 2.1. Axon model

**Figure 2:**
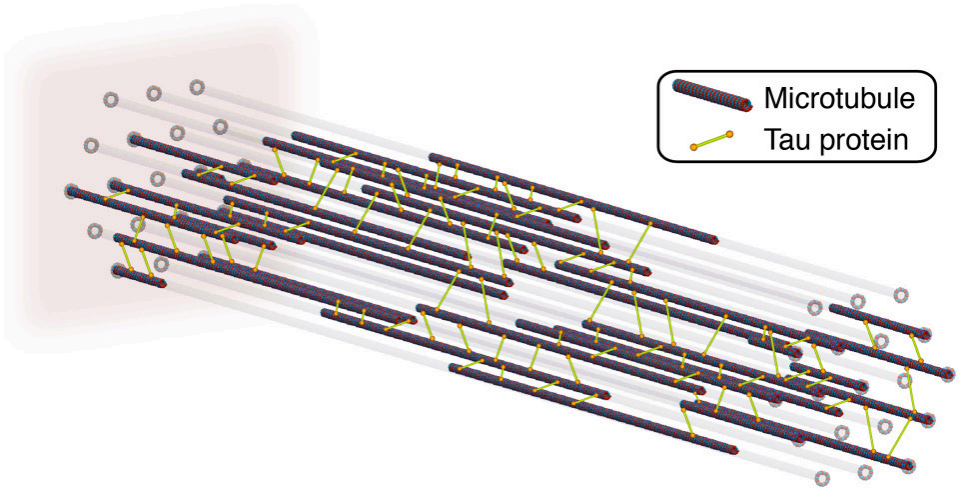
Axon model with longitudinally aligned microtubules that are connected by tau protein crosslinks. To account for axonal dynamics, we model each crosslink as a noncovalent slip bond and assign each crosslink a mechanism of Bell model type. We fix the axon at its distal, left end and apply a stretch, λ, and stretch rate, $\overset{\cdot }{\lambda }$, to its proximal, right end.

We model of the axon as a system of straight microtubules that are aligned in the longitudinal direction. Each cross section of the axon has 19 potential microtubules sites arranged in a triangular grid (de Rooij et al., 2016). On average, only half of these potential sites are occupied by a microtubule. As Figure 2 indicates, we assume that all microtubules have the same length and are *randomly* distributed along the axon. In our finite element model, each microtubule consists of 1,250 one-dimensional truss elements (de Rooij and Kuhl, 2018).

Neighboring microtubules within a cross section are crosslinked by tau protein. At the beginning of the simulation, these crosslinks are *randomly* distributed across the axon based on a crosslink density parameter. To account for the dynamic behavior of the axon, we have created an extension to the standard finite element method that can either effect individual finite elements or to sets of finite elements (de Rooij et al., 2016). This dynamic behavior represents the molecular *mechanisms* of particular proteins. Here, we assign a *mechanism* to each tau protein crosslink. The mechanism describes the dynamic behavior of crosslink detachment and reattachment by modeling the crosslink as a *slip bond*. The detachment and attachment rates, *k*(*F*), are governed by the classical Bell model (Bell, 1978) that characterizes the strength of a chemical bond under a mechanical force *F*:

(1) $$k(F)=\{\begin{array}{cc} k_{0} & \text{attach}\\ k_{0}\exp(F/F_{0}) & \text{detach} \end{array}\text{ with }F_{0}=\frac{k_{B}T}{\xi }$$

where *k*_0_ is the rate of crosslink attachment and detachment due to random thermal fluctuations. According to the Bell model for slip bonds, the likelihood of detachment increases exponentially with the physical force *F* applied to the bond. The sensitivity to this mechanical force is described by the characteristic bond strength *F*_0_ = *k*_*B*_*T*/ξ, where *k*_*B*_ is the Boltzmann constant, *T* is temperature, and ξ is the characteristic bond separation distance.

We fix the axon at its proximal end, in our model on the left side, where it connects to the cell body with the nucleus, and apply a physical stretch at the distal end, on the right side, where it connects to other axons. This implies that we apply homogeneous Dirichlet boundary conditions to the microtubules at both ends, zero on the left and non-zero on the right (de Rooij et al., 2016). We constrain all nodes in the model to move along the longitudinal axon direction only. To represent the axonal cytosol, we embed the axon in a viscous fluid with a viscosity of 5 mPa·s (Haak et al., 1976). Table 1 provides an overview of all model parameters.

**Table 1:** Parameters of the axon model, the microtubule model, and the crosslink model.

	**Value**	**Unit**	**References**
**AXON**			
Axon length	40	μm	Caminiti et al., 2013
Axon diameter	540	nm	Hirokawa, 1982
Microtubules per cross section	9.5	–	Bray and Bunge, 1981
Cytosol viscosity	5	mPa·s	Haak et al., 1976
**MIRCOTUBULES**			
Microtubule length Microtubule stiffness	1,200	MPa	Gittes et al., 1993
Microtubule area	400	nm^2^	Suresh, 2007
**CROSSLINKS**			
Crosslink distance Crosslink angle	45	deg	Hirokawa, 1982
Crosslink stiffness	10	MPa	Mallik et al., 2004
Crosslink area	1	nm^2^	de Rooij et al., 2016
Crosslink attachment rate, *k*_0_	4	1/s	Wegmann et al., 2011; Igaev et al., 2014
Crosslink bond strength, *F*_0_	1–100	pN	[varied]

*The crosslink bond strength, F_0_, is the only unknown in our model. Here, we vary the bond strength over two orders of magnitude to explore its effects on axonal damage. All other parameters are known from the literature*.

**Figure 3:**
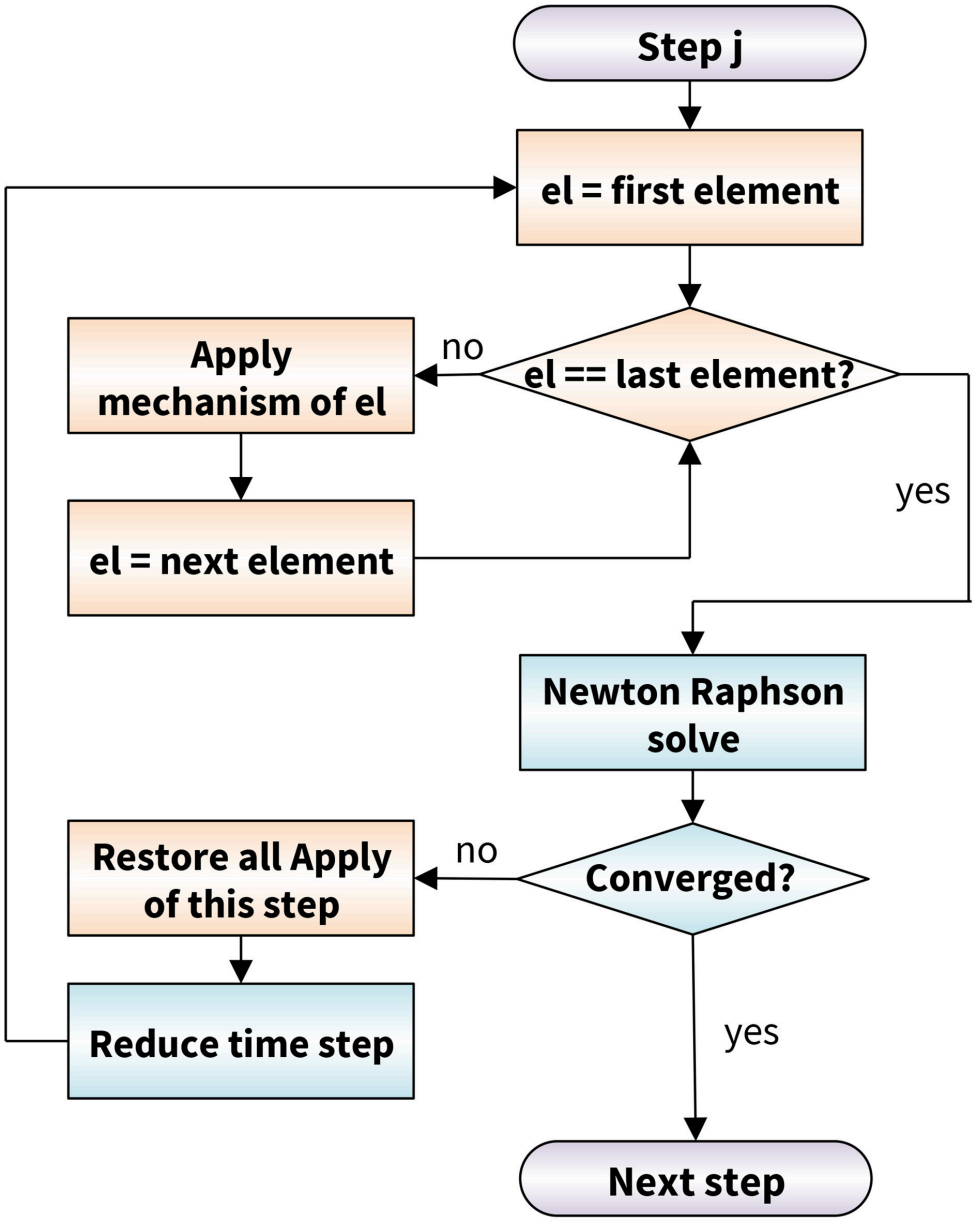
Axon model flowchart based on a conventional Newton-Raphson solver (blue) modularly enhanced by the application of mechanisms (orange). At the beginning of each Newton-Raphson step, we apply the mechanism to each crosslink element. In case the algorithms does not converge, we restore the last equilibrium state of all mechanisms and reduce the time step size.

Figure 3 illustrates the flowchart to solve our discrete axon model within our custom-designed finite element algorithm. To include the dynamic behavior of individual proteins, we extended the conventional finite element method by *mechanisms* (de Rooij et al., 2016). We include a *mechanism* by introducing a Mechanism object with an Apply() function that contains the full description of the crosslink behavior. We assign this mechanism to each crosslink and execute the Apply() function at the beginning of each iteration step in the Newton-Raphson solver with adaptive time stepping. To apply the slip bond mechanism of the Bell model, the Apply() function has to ensure that each crosslink detaches and attaches at a rate *k*(*F*) as in Equation (1) (de Rooij and Kuhl, 2018). At each time step of our simulation, we calculate the probability of detachment or reattachment. We distinguish two cases to calculate the probability of detachment: detachment at a constant force *F* and at a linearly increasing force *F* = *r*_*f*_*t*.

#### 2.1.1. Crosslink detachment at a constant force F

For a constant force *F*, we compute the probability of crosslink detachment, *p*(*F, t*), at time *t*, based on the detachment rate *k*(*F*) in Equation (1):

(2) $$\begin{array}{r} p\left(F,t\right)=k\left(F\right)\exp\left(-k\left(F\right)t\right). \end{array}$$

This probability function directly yields the probability of detachment within the next time step, between *t*_0_ and *t*_0_ + Δ*t*, as:

(3) $$P(F,\Delta t)=\frac{\int_{t_{0}}^{t_{0}+\Delta t}p(F,\bar{t})\;d\bar{t}}{\int_{t_{0}}^{\infty }p(F,\bar{t})\;d\bar{t}}=1-\exp(-k(F)\Delta t).$$

To obtain the probability of *attachment* of a crosslink, we simply substitute *F* = 0 into Equation (3).

#### 2.1.2. Crosslink detachment at a linearly increasing force *F* = *r*_f_*t*

For a linearly increasing force *F*, the probability of detachment becomes (de Rooij and Kuhl, 2018):

(4) $$\begin{array}{r} p\left(F,r_{f}\right)=\frac{k\left(F\right)}{r_{f}}\exp\left(-\frac{F_{0}}{r_{f}}\left[k\left(F\right)-k_{0}\right]\right). \end{array}$$

The probability of detachment within the next time step, between *t*_0_ and *t*_0_+Δ*t*, then follows as:

(5) $$P(F,\Delta F)=\frac{\int_{F_{0}}^{F_{0}+\Delta F}p(F)\;dF}{\int_{F_{0}}^{\infty }p(F)\;dF},$$

where Δ*F* = *r*_*f*_Δ*t*. In our simulations, we compute the individual loading rate *r*_*f*_ for each crosslink as the crosslink force divided by the time the crosslink has been attached.

### 2.2. Discrete axonal damage model

The major objective of our axon model is to interpret axonal damage as an emergent feature of the dynamic attachment and detachment of crosslinks. Motivated by the common definition of damage in continuum damage mechanics (Kachanov, 1986), we define axonal damage as the *relative loss of axonal stiffness due to excessive detachment of crosslinks as the result of a physical force*. Indeed, Equation (1) shows that a physical force *F* increases the detachment rate of crosslinks and, thereby, results in net reduction of attached crosslinks. To quantify the amount of damage, we first need to characterize the baseline, undamaged, mechanical response of our model axon. This baseline response is nonlinear, viscoelastic, and, therefore, rate dependent (de Rooij and Kuhl, 2018). We obtain the baseline response by performing simulations at an infinite characteristic bond strength *F*_0_ → ∞, see Equation (1). This implies that the detachment rate, *k*(*F*; *F*_0_ → ∞) = *k*_0_, is the same as the attachment rate, and the total number of attached crosslinks will, on average, remain constant. To account for viscous effects in the baseline response of the axon, we perform these baseline simulations for a range of loading rates *r*_*f*_.

**Figure 4:**
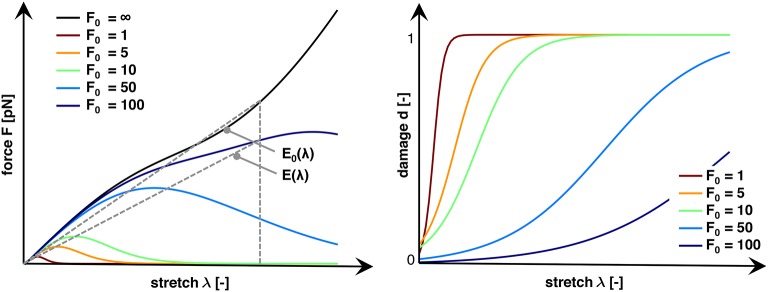
Characteristic force vs. stretch and damage vs. stretch curves for varying characteristic bond strengths, *F*_0_. An infinite bond strength *F*_0_ → ∞ defines the baseline, undamaged stiffness *E*_0_(λ). Finite bond strengths *F*_0_ trigger a net increase in crosslink detachment resulting in an increase in damage *d* and a reduced axonal stiffness *E*(λ). Increasing the bond strength *F*_0_ decreases the not crosslink detachment, which increases the stretch λ and force *F* required to trigger axonal damage. We define axonal damage, *d*(λ) = 1−*E*(λ)/*E*_0_(λ), as the ratio of the reduced and undamaged secant stiffnesses *E*(λ) and *E*_0_(λ).

We define axonal damage for a finite characteristic bond strength *F*_0_ as the relative stiffness degradation with respect to the undamaged axon. Consistent with continuum damage mechanics (Lemaitre, 1992), we use the scalar-valued damage parameter *d* to quantify axonal damage. Damage ranges from *d* = 0 for a completely intact axon to *d* = 1 for a fully damaged axon and relates the reduced stiffness *E* to the initial undamaged stiffness *E*_0_ as:

(6) $$\begin{array}{r} E=\left[1-d\right]E_{0}, \end{array}$$

where both *E* and *E*_0_ are the corresponding secant stiffnesses. Figure 4 (left) shows characteristic force-stretch curves of the axon for varying characteristic bond strengths *F*_0_. From these force-stretch curves, we compute axonal damage *d*(λ) for a given stretch λ as:

(7) $$\begin{array}{r} d\left(\lambda \right)=1-E\left(\lambda \right)/E_{0}\left(\lambda \right). \end{array}$$

Figure 4 (right) shows characteristic damage-stretch curves of the axon for varying characteristic bond strengths *F*_0_.

### 2.3. Homogenization

To bridge the scales, we postulate a specific functional form for the evolution of damage and assume that its parameters emerge naturally from the dynamic behavior of the crosslinking tau proteins. In other words, we seek an evolution equation that provides an analytical expression for axonal damage, $d\left(\lambda ,\overset{\cdot }{\lambda }\right)$, as function of the axonal stretch λ and stretch rate $\overset{\cdot }{\lambda }$. We characterize the accumulation of damage through the logistic function (Verhulst, 1845),

(8) $$d(\lambda ,\dot{\lambda })=\hat{ℋ}(\lambda -\lambda_{50}(\dot{\lambda };F_{0});\alpha (\dot{\lambda };F_{0})).$$

The *C*^∞^-smooth Heaviside function $\hat{H}\left(x;\alpha \right)=e^{\alpha x}/\left[1+e^{\alpha x}\right]$ represents an S-shaped sigmoid curve, λ_50_ is the half damage stretch at the midpoint of the S-shaped curve, at which *d*(λ = λ_50_) = 0.5, and α is proportional to the slope at this midpoint. Widely used in population dynamics, the logistic function implies that the initial stage of damage is approximately exponential, it then begins to saturate at the half damage stretch λ_50_, and gradually converges to the fully damaged state, *d* = 1, as the stretch increases. We assume that both λ_50_ and α are functions of the characteristic bond strength *F*_0_ and vary with the applied stretch rate $\overset{\cdot }{\lambda }$. Importantly, in our model, this rate dependence *emerges naturally* from the underlying crosslink dynamics.

#### 2.3.1. Ansatz for the half damage stretch $\lambda_{50}$

For the half damage stretch $\lambda_{50}\left(\overset{\cdot }{\lambda };F_{0}\right)$, we use Equation (4) to compute the expected crosslink force, $\hat{F}$, at which a crosslink detaches:

(9) $$\hat{F}=\int_{0}^{\infty }\tilde{F}p(\tilde{F};r_{f})d\tilde{F}=F_{0}\exp\left(\frac{k_{0}F_{0}}{r_{\text{​}f}}\right)\Gamma \left(0,\frac{k_{0}F_{0}}{r_{\text{​}f}}\right)$$

where $\Gamma (a,b)=\int_{b}^{\infty }e^{-x}x^{a-1}dx$ is the upper incomplete gamma function. When the crosslinks are attached to microtubules, we assume that they behave linearly elastic and we expect the transition stretch to be proportional to the detachment force, $\left[\lambda_{50}-1\right]\propto \hat{F}$. With $k_{0}F_{0}/r_{f}\propto 1/\overset{\cdot }{\lambda }$, we therefore propose:

(10) $$\begin{array}{r} \lambda_{50}\left(\overset{\cdot }{\lambda };F_{0}\right)=1+a_{\lambda }\exp\left(\frac{b_{\lambda }}{\overset{\cdot }{\lambda }}\right)\Gamma \left(0,\frac{b_{\lambda }}{\overset{\cdot }{\lambda }}\right), \end{array}$$

where *a*_λ_(*F*_0_) and *b*_λ_(*F*_0_) are parameters that depend on the characteristic bond strength *F*_0_ and will emerge naturally from the tau crosslink dynamics within the axon.

#### 2.3.2. Ansatz for the damage slope α

For the damage slope $\alpha \left(\overset{\cdot }{\lambda };F_{0}\right)$, we follow a similar approach. Since we interpret damage as the net loss of crosslinks, axonal damage is a function of the fraction of attached crosslinks ${\hat{n}}_{\text{att}}$:

(11) $$\begin{array}{r} d=1-{\hat{n}}_{\text{att}}\;\text{with}\;{\hat{n}}_{\text{att}}=\frac{{\hat{t}}_{\text{att}}}{\frac{1}{2}\left[{\hat{t}}_{\text{att}}+{\hat{t}}_{\text{det}}\right]} \end{array}$$

where ${\hat{t}}_{\text{att}}$ and ${\hat{t}}_{\text{det}}$ are the expected duration that a crosslink is attached and detached respectively. This implies that, in the limit of homeostasis between attachment and detachment, ${\hat{t}}_{\text{att}}={\hat{t}}_{\text{det}}$ and ${\hat{n}}_{\text{att}}=1$ and *d* = 0, whereas in the limit of an excessive detachment, ${\hat{t}}_{\text{att}}<<{\hat{t}}_{\text{det}}$ and ${\hat{n}}_{\text{att}}=0$ and *d* = 1. From the definition of the S-curve, we know that α is proportional to the slope of the damage curve at *d* = 0.5:

(12) $$\alpha \propto {\frac{\text{d}d}{\text{d}\lambda }|}_{d=0.5}={\frac{\text{d}d}{\text{d}{\hat{t}}_{\text{att}}}\cdot \frac{\text{d}{\hat{t}}_{\text{att}}}{\text{d}\dot{\lambda }}\cdot \frac{\text{d}\dot{\lambda }}{\text{d}\lambda }|}_{\text{d}=0.5}\propto \frac{\text{d}{\hat{t}}_{\text{att}}}{\text{d}\dot{\lambda }}.$$

By combining the definition of the attachment time, ${\hat{t}}_{\text{att}}=\hat{F}/r_{f}$, with Equations (9) and (12), we propose:

(13) $$\begin{array}{r} \alpha \left(\overset{\cdot }{\lambda };F_{0}\right)=\frac{a_{\alpha }}{{\left(\overset{\cdot }{\lambda }\right)}^{3}}\left[\overset{\cdot }{\lambda }-\left[b_{\alpha }+\overset{\cdot }{\lambda }\right]\exp\left(\frac{b_{\alpha }}{\overset{\cdot }{\lambda }}\right)\Gamma \left(0,\frac{b_{\alpha }}{\overset{\cdot }{\lambda }}\right)\right], \end{array}$$

where, again, *a*_α_(*F*_0_) and *b*_α_(*F*_0_) are parameters that depend on the characteristic bond strength *F*_0_ and will emerge naturally from the tau crosslink dynamics within the axon.

### 2.4. Continuum axonal damage model

To embed the homogenized equations into a continuum axonal damage model, we introduce the deformation φ(*X, t*) along the axis of the axon and define the axon level stretch λ and stretch rate $\overset{\cdot }{\lambda }$,

(14) $$\begin{array}{r} \lambda =\frac{\partial \varphi \left(X,t\right)}{\partial X}\;\text{and }\overset{\cdot }{\lambda }=\frac{\text{d}\lambda \left(X,t\right)}{\text{d}t}. \end{array}$$

We then introduce the free energy density of the damaged axon Ψ as the damage weighted stored energy of the undamaged, elastic axon Ψ_0_,

(15) $$\Psi (\lambda ,\dot{\lambda })=[1-d]\;\Psi_{0}(\lambda )\text{ with }d=d(\lambda ,\dot{\lambda }),$$

and assume that the evolution of damage is driven by both stretch and stretch rate, $d\left(\lambda ,\overset{\cdot }{\lambda }\right)$, while the elastic energy is a function of the stretch alone Ψ_0_(λ). Motivated by standard arguments of thermodynamics, we introduce the Cauchy stress σ = *Pλ* and the Piola stress *P* as thermodynamically conjugate quantity to the stretch λ, and interpret the Piola stretch *P* as damage weighted elastic axonal stress *P*_0_,

(16) $$\begin{array}{r} P=\frac{\partial \Psi }{\partial \lambda }=\left[1-d\right]P_{0}\;\text{with }P_{0}=\frac{\partial \Psi_{0}}{\partial \lambda }. \end{array}$$

To keep track of the maximum amount of stretch the axon has experienced throughout its history, it is common practice to introduce an internal variable,

(17) $$\begin{array}{r} \lambda^{*}=\underset{0\leq t\leq \tau }{\max}\left\{\lambda \left(t\right)\right\}, \end{array}$$

which drives the evolution of damage,

(18) $$\begin{array}{r} d=\frac{\exp\left(\alpha \left[\lambda^{*}-\lambda_{50}\right]\right)}{1+\exp\left(\alpha \left[\lambda^{*}-\lambda_{50}\right]\right)}. \end{array}$$

The stretch rate dependent half damage stretch,

(19) $$\begin{array}{r} \lambda_{50}\left(\overset{\cdot }{\lambda };F_{0}\right)=1+a_{\lambda }\exp\left(\frac{b_{\lambda }}{\overset{\cdot }{\lambda }}\right)\Gamma \left(0,\frac{b_{\lambda }}{\overset{\cdot }{\lambda }}\right), \end{array}$$

and the stretch rate dependent damage slope,

(20) $$\begin{array}{r} \alpha \left(\overset{\cdot }{\lambda };F_{0}\right)=\frac{a_{\alpha }}{{\left(\overset{\cdot }{\lambda }\right)}^{3}}\left[\overset{\cdot }{\lambda }-\left[b_{\alpha }+\overset{\cdot }{\lambda }\right]\exp\left(\frac{b_{\alpha }}{\overset{\cdot }{\lambda }}\right)\Gamma \left(0,\frac{b_{\alpha }}{\overset{\cdot }{\lambda }}\right)\right], \end{array}$$

follow from the homogenization in section 2.3 and vary with the bond strength *F*_0_ of the individual crosslinks in the axon. Last, to solve the continuum equations of axonal damage within a finite element setting, we derive the tangent modulus,

(21) $$\begin{array}{r} \text{A}=\frac{\text{d}P}{\text{d}\lambda }=\left[1-d\right]{\text{A}}_{0}-\frac{\text{d}d}{\text{d}\lambda }P_{0}\;\text{with }{\text{A}}_{0}=\frac{\text{d}P_{0}}{\text{d}\lambda }. \end{array}$$

For our specific damage model with

(22) $$\begin{array}{ll} & \frac{\text{d}d}{\text{d}\lambda }=\frac{\text{d}d}{\text{d}\lambda^{*}}\frac{\text{d}\lambda^{*}}{\text{d}\lambda }\text{ with }\frac{\text{d}d}{\text{d}\lambda^{*}}=\alpha [1-\text{d}]d\\ \; & \text{and }\frac{d\lambda^{*}}{\text{d}\lambda }=\{\begin{array}{l} 1...\text{loading}\\ 0...\text{unloading} \end{array} \end{array}$$

we obtain the following simple structure of the tangent modulus,

(23) $$\begin{array}{r} \text{A}=\left[1-d\right]\left[{\text{A}}_{0}-\alpha dP_{0}\right]. \end{array}$$

For example, for an elastic material of Mooney Rivlin type, with $\Psi_{0}=c_{1}\left[\lambda^{2}+2/\lambda -3\right]+c_{2}\left[2\lambda +1/\lambda^{2}-3\right]$, the Cauchy stress becomes $\sigma =\left[1-d\right]2\left[c_{1}+c_{2}/\lambda \right]\left[\lambda^{2}-1/\lambda \right]$, the elastic tangent modulus is ${\text{A}}_{0}=2c_{1}\left[1+2/\lambda^{3}\right]+6c_{2}/\lambda^{4}$, and the elastic Piola stress is $P_{0}=2\left[c_{1}+c_{2}/\lambda \right]\left[\lambda -1/\lambda^{2}\right]$, where $c_{1}+c_{2}=\frac{1}{2}\mu$ are the two constitutive parameters of the Mooney Rivlin model and μ is the overall shear modulus of the axon (Goriely et al., 2015a).

## 3. Results

### 3.1. Axon model

**Figure 5:**
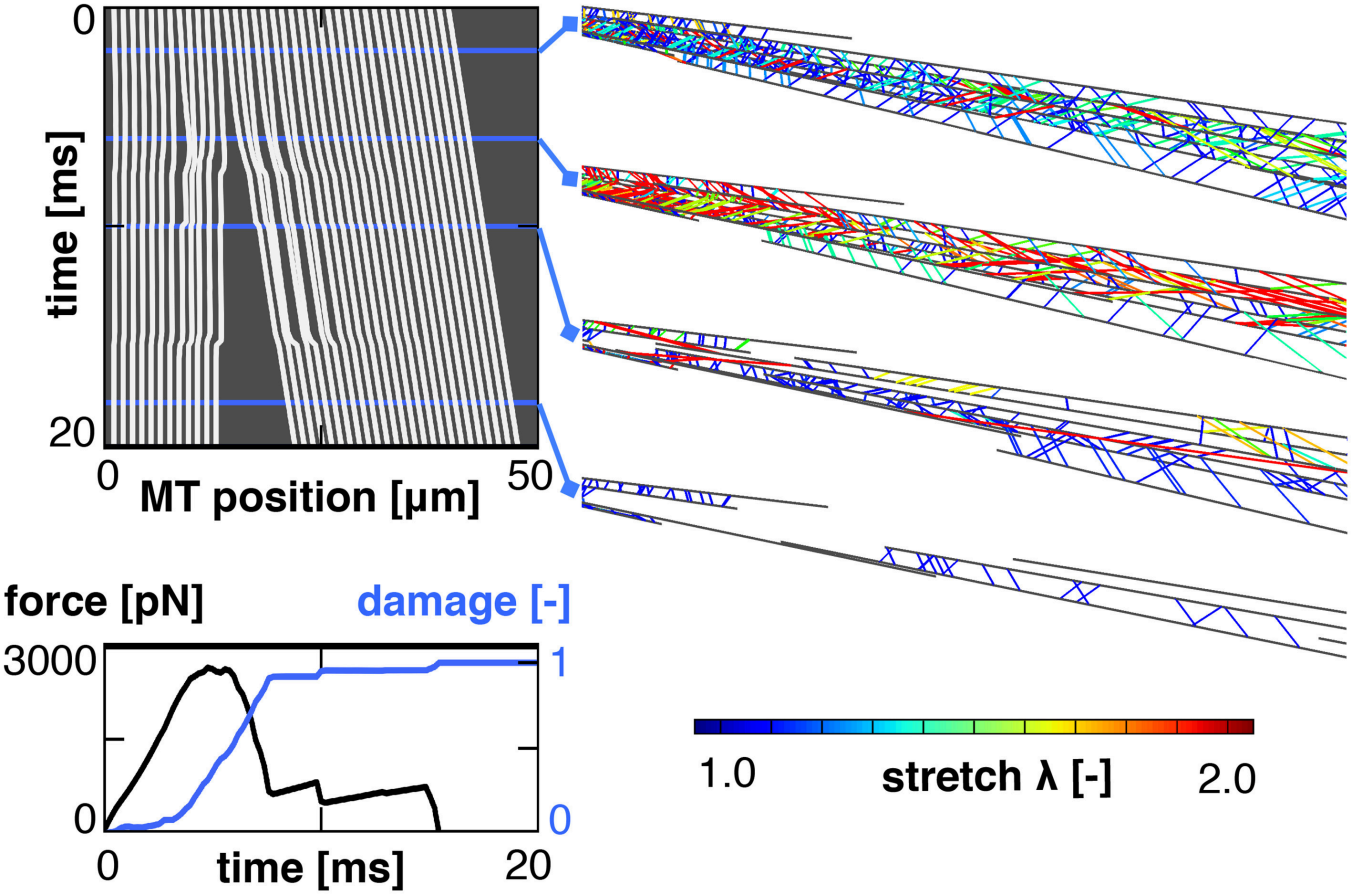
Characteristic output of a single simulation. The kymograph, **(Top left)**, tracks the position of all microtubules throughout the simulation. It reveals a complete separation between the proximal and distal ends of the axon. This separation manifests itself in a loss of the required external force and an increase in axonal damage **(Bottom left)**. Four snapshots of the axon show the crosslinks color coded by crosslink stretch **(Right)**. These snapshots show an initial increase in crosslink stretch, followed by a decrease after the proximal and distal sets of microtubule bundles have separated.

Figure 5 shows the result of a single simulation in which we applied a stretch of λ = 1.2 at a stretch rate $\overset{\cdot }{\lambda }=10$/s assuming a bond strength of *F*_0_ = 5pN.

The computational kymograph in the top left traces the location of all microtubules in the axon throughout the entire duration of the simulation. The snapshots on the top right show the axon at four different time points with the tau crosslinks color coded by the crosslink stretch. Toward the end of the simulation, the stretch in the remaining crosslinks decreases. This decrease is accompanied by a clear separation between the proximal and distal ends of the axon, as we can see from the kymograph. This separation is indicative of axonal damage; it reduces the overlap of microtubules and, thereby, the number of connecting crosslinks in the damaged region. Indeed, the force vs. time curve in the bottom left shows an initial increase in force followed by a rapid decrease approximately 5 ms into the simulation. The axonal damage, computed according to section 2.2, increases from *d* = 0 at the beginning of the simulation to *d* = 1 when the two sets of microtubule bundles are fully disconnected.

### 3.2. Discrete axonal damage

To probe the sensitivity of axonal damage with respect to stretch and stretch rates, we performed several sets of simulations for a range of stretches, λ∈[1, 1.2], and stretch rates, $\overset{\cdot }{\lambda }\in \left[0.1,10\right]$ /s. The only input parameter that is not well defined in the literature is the characteristic bond strength of the tau crosslinks. We therefore also probed a range of crosslink bond strengths, *F*_0_ = [1, 5, 10, 50, 100] pN.

**Figure 6:**
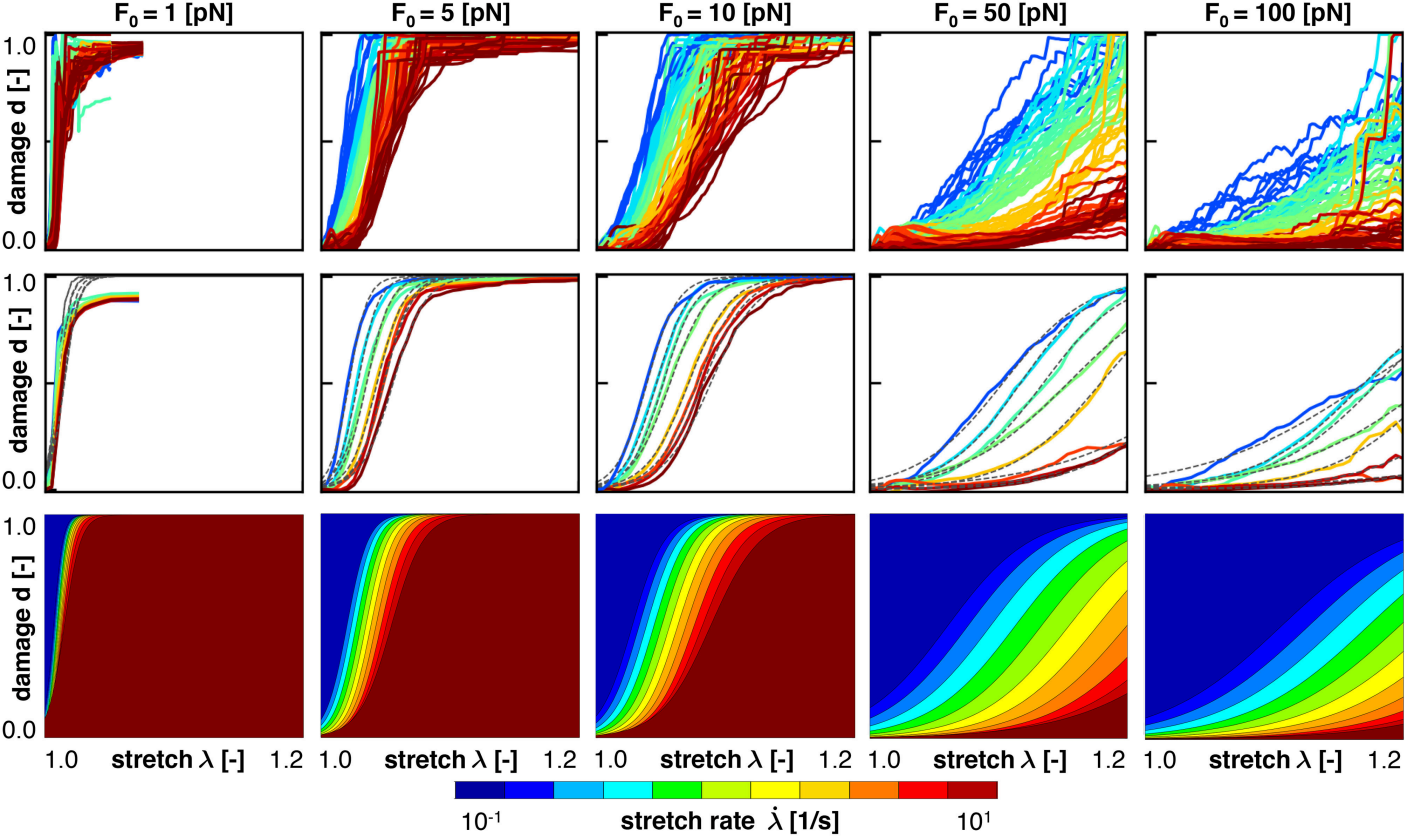
Axonal damage vs. axonal stretch color coded by the stretch rate for five bond strengths. Discrete axonal damage simulations with *n* = 10 simulations for each stretch rate **(Top)**; homogenization using the average response for each stretch rate to identify the parameters for S-shaped curve of the homogenized damage model **(Middle)**; and continuum axonal damage simulation **(Bottom)**. The graphs demonstrate an important feature of the Bell model: at higher stretch rates, axonal damage occurs at higher axonal stretch.

Figure 6 (top) shows the damage vs. stretch curves for *n* = 1,000 simulations at all five crosslink bond strengths, *F*_0_ = [1, 5, 10, 50, 100] pN, color coded by the stretch rate $\overset{\cdot }{\lambda }\in \left[0.1,10\right]$ /s. Consistent with the Bell model, for smaller values of *F*_0_, axonal damage develops earlier, at lower axonal stretch levels, than for larger values of *F*_0_. Indeed, Equation (1) shows that at lower crosslink bond strengths *F*_0_, the detachment rates for a given applied crosslink force *F* are higher, which manifests itself in an increased axonal damage. Our axon model also predicts that crosslink detachment is more likely to happen at higher crosslink stretches for high loading rates, which follows directly from Equation (9). Figure 6 captures this prediction as axonal damage develops at higher values for axonal stretch for high loading rates. Interestingly, this trend is reversed when considering damage vs. *loading time*.

**Figure 7:**
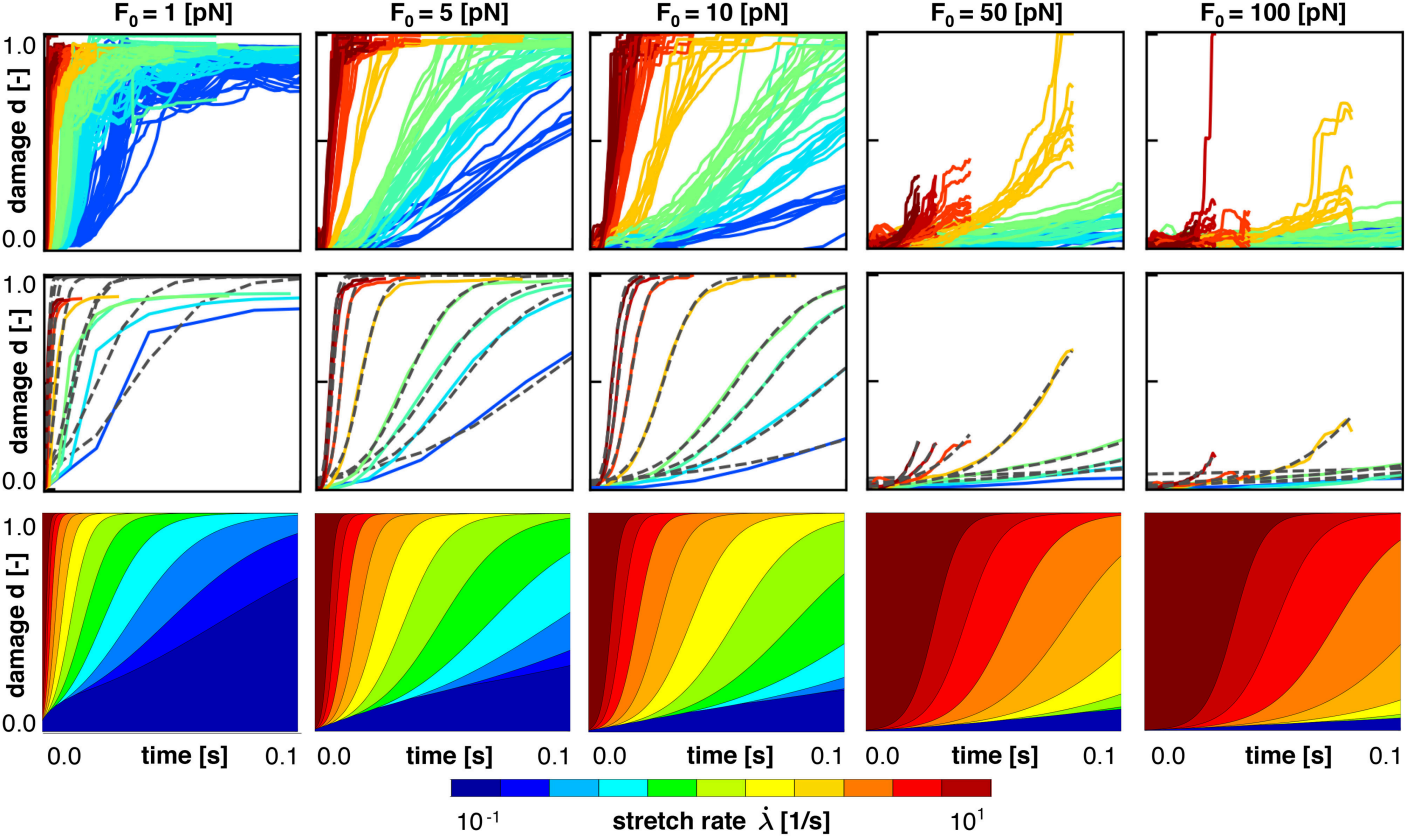
Axonal damage vs. time color coded by the stretch rate for five bond strengths. Discrete axonal damage simulations with *n* = 10 simulations for each stretch rate **(Top)**; homogenization using the average response for each stretch rate to identify the parameters for S-shaped curve of the homogenized damage model **(Middle)**; and continuum axonal damage simulation **(Bottom)**. The graphs represent the same data as in Figure 6, and demonstrate an important feature of the Bell model: at higher stretch rates, axonal damage occurs at higher axonal stretch, but earlier in time.

Figure 7 shows the same simulations as Figure 6, but now as damage plotted vs. loading time. It is clearly visible that higher applied stretch rates lead to *earlier* development of axonal damage. Thus, increased loading rates triggers *earlier* development of axonal damage, but at a *higher* axonal stretch, all consistent with the Bell model.

### 3.3. Homogenization

To homogenize the results or our discrete axon model simulation toward an overall constitutive damage model for the axon, we calibrate our damage model $d\left(\lambda ,\overset{\cdot }{\lambda }\right)$ in Equations (8), (19), and (20), using our discrete axon level simulations. For each characteristic crosslink force and applied stretch rate, we compute the *mean* damage vs. stretch curve from our simulations. For each mean curve, we calibrate the half damage stretch λ_50_ and the damage slope α according to Equation (8). Figures 6, 7 (middle) illustrate the mean damage curves for each applied stretch rate, together with the best analytical fit as a dashed, black line. These figures show a good qualitative agreement between the discrete axon model and its homogenized response, which supports our initial selection for the damage evolution law, $d\left(\lambda ,\overset{\cdot }{\lambda }\right)$, in Equation (8).

For each applied stretch rate, $\overset{\cdot }{\lambda }$, the homogenization yields one value for the half damage stretch λ_50_ and for the damage slope α, assuming a fixed characteristic bond strength, *F*_0_. In the next step, we use these values together with Equations (19) and (20) to obtain a stretch-rate dependent half damage stretch $\lambda_{50}\left(\overset{\cdot }{\lambda }\right)$ and damage slope $\alpha \left(\overset{\cdot }{\lambda }\right)$. From the best fits, we obtain discrete values for the parameters *a*_λ_ and *b*_λ_ in Equation (19) and *a*_α_ and *b*_α_ in Equation (20). Naturally, these four parameters will be different for different characteristic bond strengths *F*_0_.

**Figure 8:**
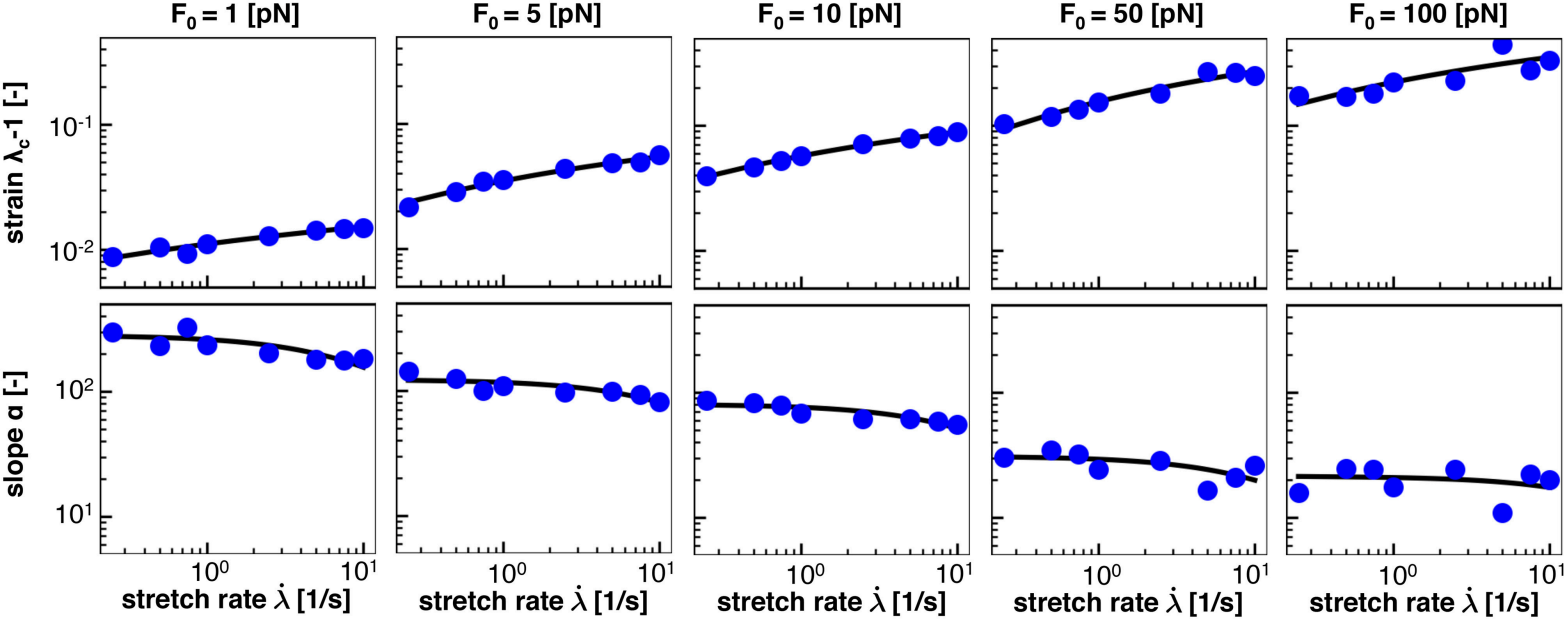
Homogenization of half damage stretch λ_50_ and damage slope α from average response of *n* = 10 discrete axonal damage simulations for as function of the stretch rate, $\overset{\cdot }{\lambda }$, for five bond strengths, *F*_0_. Blue dots represents λ_50_ and α values from the average damage vs. stretch relations in Figure 6; solid black lines represent homogenization using Equations (19) and (20) for five bond strengths, *F*_0_.

Figure 8 shows the numerical data points and the analytical fits for the half damage stretch $\lambda_{50}\left(\overset{\cdot }{\lambda }\right)$ and for the damage slope $\alpha \left(\overset{\cdot }{\lambda }\right)$ for the range of *F*_0_ = [1, 5, 10, 50, 100] pN. Qualitatively, Figure 8 confirms that our expressions for $\lambda_{50}\left(\overset{\cdot }{\lambda };F_{0}\right)$ and $\alpha \left(\overset{\cdot }{\lambda };F_{0}\right)$ in Equations (19) and (20), respectively, accurately represent the simulation data. In addition, Figure 8 confirms that λ_50_ increases with increasing stretch rate, $\overset{\cdot }{\lambda }$, and with increasing crosslink bond strength, *F*_0_. Both trends are consistent with our crosslink model and with Figure 6. In contrast, the damage slope parameter α decreases with increasing stretch rate, $\overset{\cdot }{\lambda }$, and with increasing crosslink bond strength, *F*_0_.

Table 2 summarizes the homogenized parameters *a*_λ_, *b*_λ_, *a*_α_, and *b*_α_, for bond strengths within the range *F*_0_ = [1, 5, 10, 50, 100] pN. With increasing bond strength *F*_0_, *a*_λ_, *b*_λ_, and *b*_α_ increase, while *a*_α_ decreases. To interpolate between the five bond strengths, we suggest the following rationale: Motivated by Equations (19) and (20), we expect that *a*_λ_ → 0 and *a*_α_ → ∞ as the characteristic bond strength decreases toward zero, *F*_0_ → 0. This suggests power law relations for the parameters *a* of the form $a_{\lambda }=1.936\cdot 10^{-3}{\left(F_{0}\right)}^{0.835}$ and $a_{\alpha }=-0.675{\left(F_{0}\right)}^{-0.166}$. Motivated by phenomenological considerations, for the parameters *b*, we suggest a linear dependence on ln(*F*_0_) of the form $b_{\lambda }=4.64\cdot 10^{-6}\ln\left(F_{0}\right)+1.61\cdot 10^{-6}$ and $b_{\alpha }=1.55\cdot 10^{-2}\ln\left(F_{0}\right)+4.55\cdot 10^{-2}$. This completes our damage model that is fully determined as function of stretch, λ, and stretch rate $\overset{\cdot }{\lambda }$, parameterized by the characteristic bond strength, *F*_0_.

**Table 2:** Homogenized axon parameters *a*_λ_ and *b*_λ_ to calculate the half damage stretch λ_50_ according to Equation (19) and *a*_α_ and *b*_α_ to calculate the damage slope α according to Equation (20) for a range of characteristic bond strengths *F*_0_.

**Parameter**	***F*_0_ = 1 pN**	***F*_0_ = 5 pN**	***F*_0_ = 10 pN**	***F*_0_ = 50 pN**	***F*_0_ = 100 pN**
*a*_λ_ [-]	1.81·10^−3^	8.74·10^−3^	1.40·10^−2^	5.26·10^−2^	5.76·10^−2^
*b*_λ_ [1/ms]	1.28·10^−6^	1.11·10^−5^	1.01·10^−5^	3.40·10^−5^	1.28·10^−5^
*a*_α_ [-]	−0.655	−0.675	−0.384	−0.151	−0.633
*b*_α_ [1/ms]	0.048	0.074	0.069	0.070	0.1723

### 3.4. Continuum axonal damage

Once homogenized and calibrated, we can use the axonal damage model and embed it into a continuum damage simulation using the governing equations from section 2.4. We can, for example, embed these equations into a nonlinear finite element analysis and project the homogenized axonal response along the axonal direction, in a one-, two-, or fully three-dimensional brain model. These continuum damage simulations are fully determined by our evolution equations for the damage variables $d\left(\lambda ,\overset{\cdot }{\lambda }\right)$, which emerge naturally from the axon-level crosslink dynamics.

Figures 6, 7 (bottom) summarize the resulting damage vs. stretch and damage vs. time contours color coded by the stretch rate for five bond strengths. Both graphs highlight an important feature of the Bell model: at higher stretch rates, axonal damage occurs at higher axonal stretch, but earlier in time. Comparing the continuum axonal damage model (bottom) to the discrete axonal damage model (top) and its homogenization (middle) confirms that our transient damage model at the continuum level captures the same failure characteristics as the discrete axonal damage model based on crosslink detachment and reattachment dynamics.

**Figure 9:**
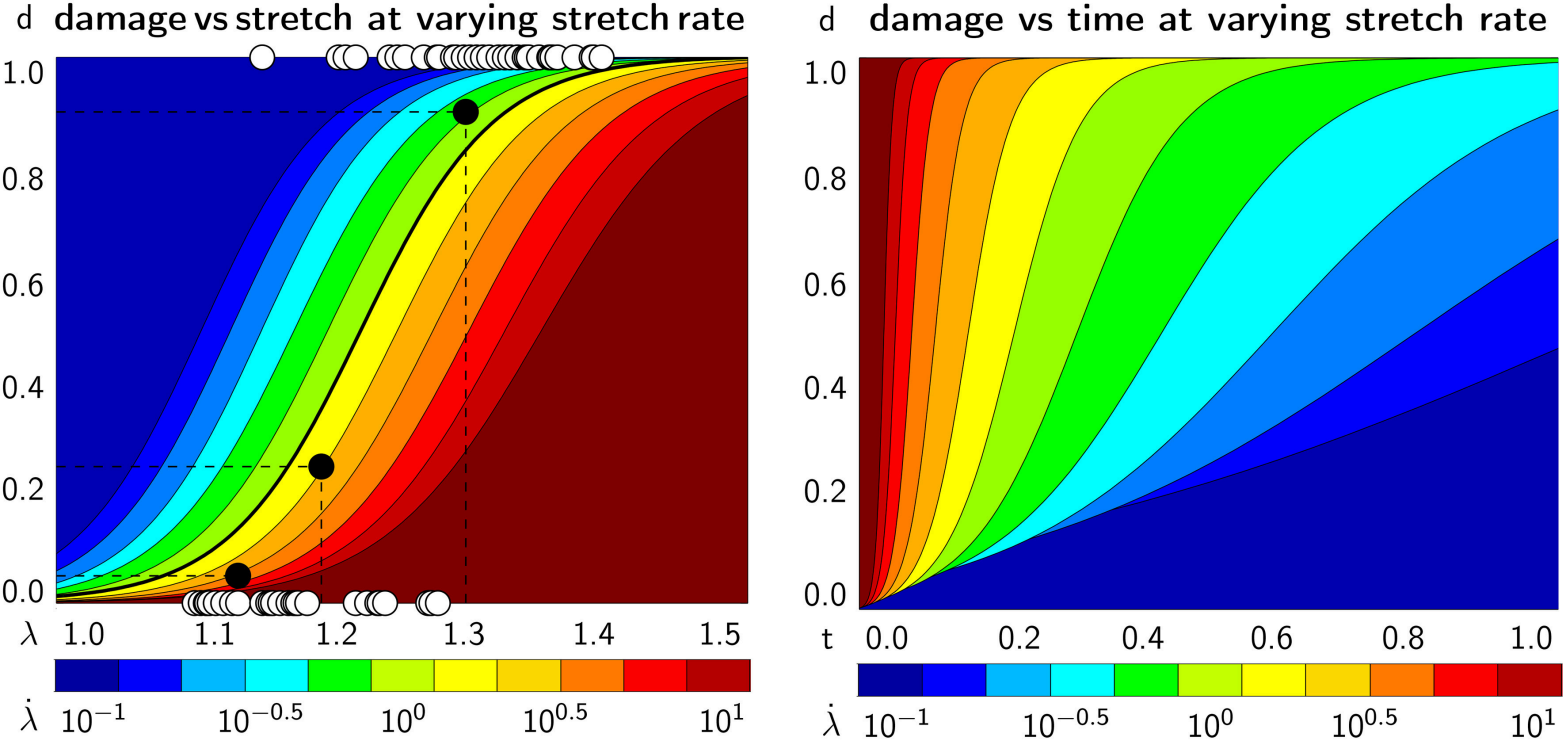
Continuum axonal damage. Axonal damage vs. axonal stretch **(Left)** and time **(Right)** at varying stretch rates and constant bond strength. White circles represent experimentally characterized damaged, *d* = 1, and undamaged, *d* = 0, nervous tissue samples at different strain levels (Bain and Meaney, 2000). Black circles define the conservative damage threshold at 14% strain, the liberal damage threshold at 34% strain, and the optimal damage threshold at 21% strain (Bain and Meaney, 2000). The graphs demonstrate an emergent feature of our transient crosslink model: For increasing stretch rates, damage develops at a larger stretch **(Left)** but earlier in time **(Right)**.

Figure 9 illustrates the emergent axonal damage vs. stretch λ[1.0, 1.5] (left) and time *t*∈[0.0, 1.0] s (right) at varying stretch rates $\overset{\cdot }{\lambda }\in \left[0.1,10\right]$/s at a constant bond strength of *F*_0_ = 100 pN. The white circles represent experimentally characterized damaged, *d* = 1, and undamaged, *d* = 0, nervous tissue samples that had been exposed to different strain levels (Bain and Meaney, 2000). These experiments clearly report a transition from a low likelihood of damage at low stretch rates to a high likelihood of damage at high stretch rates and characterize the critical stretch levels at which damage emerges. The black circles define the conservative damage threshold at *d* = 0.05 for 14% strain and a stretch of 1.13, the liberal damage threshold at *d* = 0.90 for 34% strain and a stretch of 1.30, and the optimal damage threshold at *d* = 0.25 for 21% strain and a stretch of 1.19, indicated through the black dashed dlines (Bain and Meaney, 2000). The thick black line highlights the best fit. Its half damage stretch is λ_50_ = 1.22, which implies that at a stretch of 1.22 corresponding to a 25% strain, half of the samples were damaged. The graphs in Figure 9 demonstrate an emergent feature of our transient crosslink model: For increasing stretch rates, damage develops at a larger stretch (left) but earlier in time (right).

**Figure 10:**
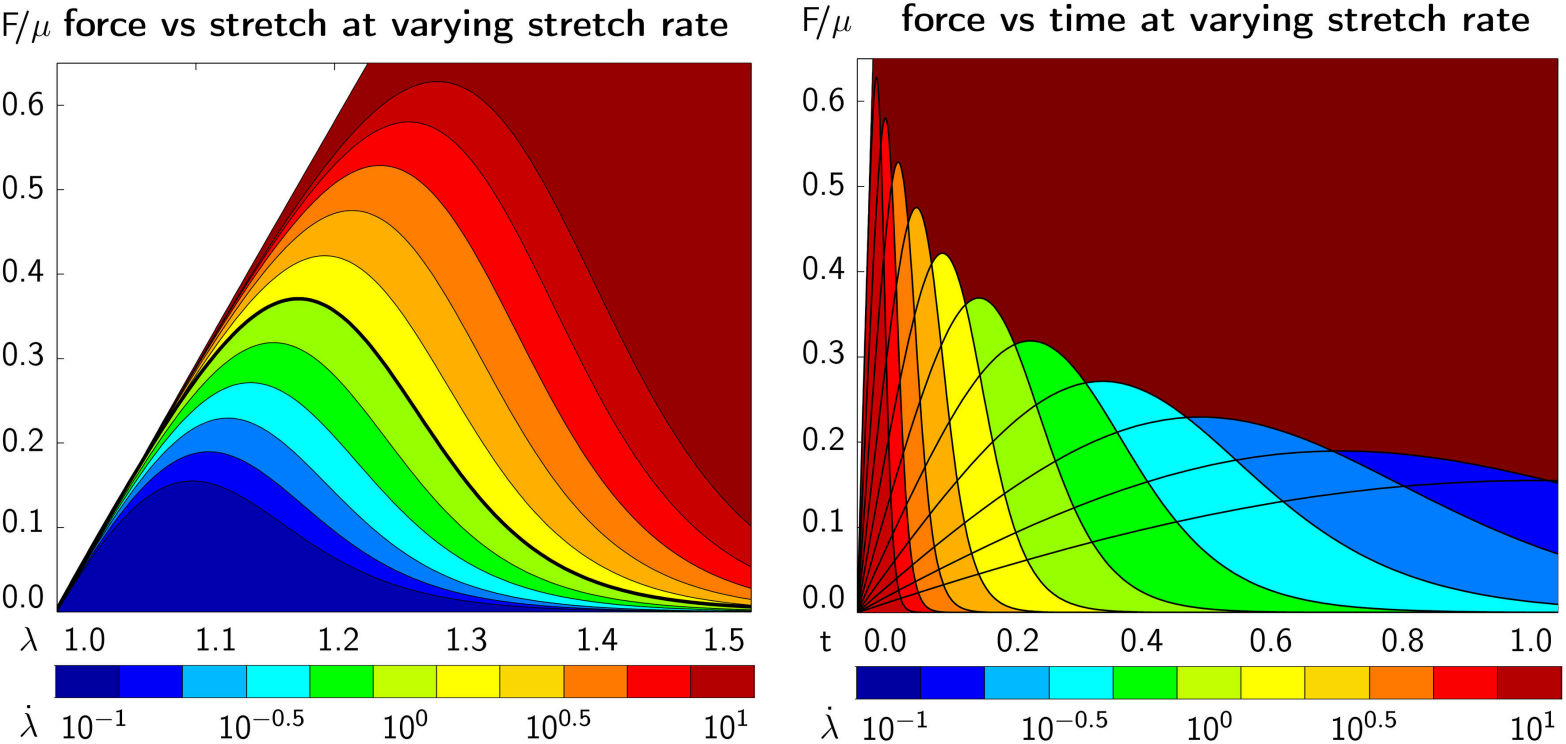
Continuum axonal damage. Axonal force vs. axonal stretch **(Left)** and time **(Right)** at varying stretch rates and constant bond strength. The dark red area marks the elastic, undamaged regime. The graphs demonstrate an emergent feature of our transient crosslink model: For increasing stretch rates, the peak axonal force increases **(Left)** but is reached earlier in time **(Right)**.

Figure 10 illustrates the emergent axonal force vs. stretch λ∈[1.0, 1.5] (left) and time *t*∈[0.0, 1.0] s (right) at varying stretch rates $\overset{\cdot }{\lambda }\in \left[0.1,10\right]$/s at a constant bond strength of *F*_0_ = 100 pN. The dark red area marks the elastic, undamaged regime, here for the example of a Mooney Rivlin model according to section 2.4, with a parameter ratio of *c*_1_:*c*_2_ = 3:1. All other colors highlight the effect of damage with a gradually increasing force that eventually reaches a peak and decreases as a result of axonal softening. The continuum level force vs. time behavior (right) agrees well with the axon level force vs. time behavior in Figure 5 (bottom left). The graphs in Figure 10 demonstrate an emergent feature of our transient crosslink model: For increasing stretch rates, the peak axonal force increases (left) but is reached earlier in time (right).

## 4. Discussion

Brain damage can be caused by a wide variety of physical impacts, ranging from a single and strong blow to the head to several mild but repeated concussive events. Independent of the type of impact, brain damage typically originates at the level of the axon: Diffuse axonal injury may develop *instantaneously* upon a single severe impact, whereas chronic traumatic encephalopathy develops *gradually* in response to repeated mild impacts to the head. Yet, the precise mechanisms how physical impacts to the head triggers pathologies at the axon level remain incompletely understood (Goriely et al., 2015b). With current technologies, we cannot reliably measure the direct effects of physical forces to the head. However, mechanical modeling can help to indirectly assess the effects of physical impact and correlate external loading to critical damage thresholds on the axonal level (Greenwald et al., 2008; Giordano et al., 2017; Kuo et al., 2017). Toward this goal we propose a mechanistic biophysical model that interprets axonal damage as an emergent property of crosslink dynamics, physical stretches, and stretch rates.

### 4.1. Axonal damage is a result of excessive crosslink detachment

We model the axon as a parallel arrangement of longitudinally aligned microtubules that are crosslinked by tau protein. These crosslinks can break and form according to the Bell model for chemical bond breaking (Bell, 1978) under external physical forces (Evans and Ritchie, 1997). The Bell model is characterized by a characteristic bond strength, *F*_0_, which is the only unknown variable in our axon model. All other parameters in the model have been reported in the literature as summarized in Table 1. To investigate the evolution of damage inside the axon, we apply a displacement controlled external stretch at different stretch rates. Damage emerges naturally as a result of the stretch-induced forces acting on the crosslinks, which, according to the Bell model, triggers a net increase of crosslink detachment. From a physics perspective, we define axonal damage as the loss in axon stiffness (Kachanov, 1986; Lemaitre, 1992) triggered by a gradual loss of crosslinks (Ahmadzadeh et al., 2014) that promotes microtubule depolymerization (Kadavath et al., 2015) and destabilizes the axonal cytoskeleton and (Krieg et al., 2017).

### 4.2. Damage accumulates at the location of weakest connectivity

A representative simulation of axonal damage is characterized by an applied stretch, λ, at a given stretch rate, $\overset{\cdot }{\lambda }$. The main output of our simulation is the overall force-stretch behavior of the axon, see Figure 5. We use this axonal force-stretch response to derive the axonal damage-stretch response compared to the undamaged baseline case, see section 2.2. Figure 5 shows that the axonal force increases initially, peaks, and then quickly drops down to zero. This drop is a defining feature of axonal damage and indicates that the axon has lost all its mechanical resistance to loading. The kymograph in Figure 5 illustrates that this rapid loss in stiffness is associated with a *primary axotomy*, the development of two disconnected sets of microtubule bundles, one at the proximal and one at the distal end, which ultimately defines axonal failure. The exact location of the axotomy is stochastic due to the probabilistic nature of the slip bond model and the underlying axon geometry: Once a weak cross section *randomly* develops along the axon, each remaining crosslinks in this cross section has to carry more mechanical load; this increases its probability of detachment, which increases the probability that the cross section becomes even weaker and eventually fails completely.

### 4.3. At higher stretch rates, axons can sustain higher stretches

We perform numerical simulations for a range of stretches λ∈[1, 1.2] and stretch rates $\overset{\cdot }{\lambda }\in$ [0.1,10] /s, and systematically vary the characteristic bond strength over two orders of magnitude *F*_0_∈ [1,100] pN. Motivated by the randomness in the precise axonal geometry and in the time of detachment and attachment of individual crosslinks, we perform *n* = 10 simulations for each set of input parameters and use the average result of those simulations for further analysis. Figures 6, 7 (top) show the axonal damage vs. stretch and time for a range of stretch rates. The results in these figures are consistent with an important emergent feature of our transient crosslink model: at *higher* applied stretch rates, axonal damage develops at a *higher* axonal stretch, but *earlier* in time (de Rooij and Kuhl, 2018).

### 4.4. The homogenized axonal damage behavior displays an S-shaped response

To derive a constitutive model for axonal damage that we can embed into whole brain damage simulations (Goriely et al., 2015a), we homogenize the discrete axonal response. For each combination of stretch, stretch rate, and characteristic bond strength, we perform *n* = 10 discrete axonal damage simulations and average their damage-stretch response. We homogenize the discrete model by fitting an S-shaped damage curve through the average damage-stretch response. The S-shaped damage model is similar to damage evolution laws for soft materials that exponentially approach complete damage at *d* = 1 (Beatty and Krishnaswamy, 2000; Weisbecker et al., 2012). However, several damage evolution laws assume that damage only starts beyond a certain stretch threshold (Peerlings et al., 2001). Here, we choose the *smooth* S-shaped curve because our crosslink model is also *smooth* (Bell, 1978) and because it nicely captures the homogenized axonal response in Figures 6, 7 (middle).

### 4.5. The damage-stretch behavior of the axon is defined by two parameters

Our S-shaped damage curve is defined by two parameters with a clear physical interpretation: the half damage stretch λ_50_ that defines the stretch at which the axon is half damaged and the damage slope α associated with the slope at this half damage stretch. Note that the half damage stretch λ_50_ is conceptually similar to the damage stretch threshold that has been proposed in literature (Marini et al., 2012; Li, 2016). We assume that both the half damage stretch and the damage slope depend on stretch rate and characteristic crosslink force, $\lambda_{50}\left(\overset{\cdot }{\lambda };F_{0}\right)$ and $\alpha \left(\overset{\cdot }{\lambda };F_{0}\right)$. We derive the qualitative dependence on the stretch rate $\overset{\cdot }{\lambda }$ from the Bell model in Equations (19) and (20), which we compare to our discrete axon simulations in Figure 8. Figure 8 reveals that the half damage stretch, λ_50_, increases with increasing stretch rate and with increasing bond strength. The increase of λ_50_ with increasing stretch rate $\overset{\cdot }{\lambda }$ is consistent with the Bell model that assumes that, at higher stretch rates, the axon can sustain higher stretches prior to damaging. The increase of λ_50_ with increasing bond strength *F*_0_ is also consistent with the Bell model in Equation (1), since higher bond strengths *F*_0_ require higher forces *F* to generate the same detachment rate. Figure 8 shows that the slope, α, decreases with increasing stretch rate and increasing bond strength. This is consistent with the Bell model in Equation (20) and with the smoothness of the Bell model. Table 2 summarizes the homogenized axon parameters *a*_λ_ and *b*_λ_ to calculate the half damage stretch λ_50_ and the homogenized axon parameters *a*_α_ and *b*_α_ to calculate the damage slope α for a range of bond strengths *F*_0_. Table 2 suggests that, with increasing bond strength *F*_0_, *a*_λ_, *b*_λ_, and *b*_α_ increase while *a*_α_ decreases.

### 4.6. Our damage model agrees well with experimental findings

Section 2.4 and Figures 6, 7 (bottom) summarize our constitutive model for axonal damage. Our damage model is completely determined as a function of stretch λ and stretch rate $\overset{\cdot }{\lambda }$, parameterized in the bond strength *F*_0_. Although the characteristic bond strength *F*_0_ is currently unknown, our selected range *F*_0_∈ [1,100] pN lies well within the range of physiological force levels observed at the protein level. For example, dynein protein generates a force of about 1 pN (Mallik et al., 2004), microtubule assembly may generate pushing forces up to 3–4 pN (Dogterom and Yurke, 1997), and the growth cone of the axon generates a total pulling force of about 1–20 nN (Rajagopalan et al., 2010; Hyland et al., 2014). Strikingly, Figure 8 suggests that the values for the half damage stretch λ_50_ all lie within λ_50_∈ [1.01,1.3] for our entire range of stretch rates $\overset{\cdot }{\lambda }\in$ [0.1,10] /s and bond strengths *F*_0_∈ [1,100] pN. By definition, λ_50_ is the axonal stretch at which 50% of the axon is damaged. This suggests that we can use the λ_50_ value as a surrogate measure for the axonal damage level threshold. The range of λ_50_∈ [1.01,1.30] agrees well with reported critical stretch values for axonal injury: critical values between 1.05 and 1.10 have been found based on animal and physical studies (Margulies and Thibault, 1992), a critical stretch of 1.05 caused mild injury in cortical axons in culture (Yuen et al., 2009), axonal stretch between 1.09 and 1.16 led to axonal injury in rats (Singh et al., 2016), and critical stretches between 1.14 and 1.34 were found at stretch rates between 30 and 60 /s in white matter brain tissue (Bain and Meaney, 2000). Notably, these reported critical damage stretch thresholds are all based on a single severe loading of the axon and mimic the event of traumatic brain injury. To date, there is no reliable data on critical damage stretch thresholds for multiple mild loading of the axon that would mimic the event of chronic traumatic encephalopahy (Asken et al., 2017).

### 4.7. Our damage model integrates well into finite element algorithms

Once calibrated and validated, we can embed our constitutive model for axonal damage in a continuum mechanics model for whole brain simulations and superpose it to the isotropic behavior of the tissue (Cloots et al., 2013; Mao et al., 2013; Goriely et al., 2015b; Giordano et al., 2017; Weickenmeier et al., 2017). Figures 9, 10 show that the continuum implementation of the damage model, e.g., within a nonlinear finite element setting, correctly reproduces the axon level features of damage. Notably, a well-known problem with continuum damage models is that, in the softening regime, the governing equations become ill posed and the numerical solutions become mesh dependent. These issues can be resolved with appropriate regularization techniques (de Borst et al., 1993; Kuhl et al., 2000). A natural regularization technique is to account for the rate dependent nature of damage (Geers et al., 1994). Although we do not explicitly investigate regularization here, the inherent rate dependence of our axonal damage model potentially regularizes the simulation at no additional cost (Pereira et al., 2017).

### 4.8. Our axon model has a few limitations

We have proposed a consistent strategy to relate microscale protein behavior to axonal damage and to develop a constitutive damage model that can be used at the continuum, whole brain level. However, we do recognize several limitations to our model that we plan to address in future work: First, our model assumes that axonal damage is solely caused by the disruption in tau protein crosslinks. Although this disruption is consistent with, e.g., the diagnoses of chronic traumatic encephalopathy through an abnormal increase of unbound tau, other mechanisms such as microtubule rupture may contribute to damage of the axon (Tang-Schomer et al., 2010; van den Bedem and Kuhl, 2015). In addition, we simplified the axonal cytoskeleton as a composition of microtubules and tau proteins, while we recognize that the axon contains additional cytoskeletal elements and organelles that could be structurally relevant, such as neurofilaments, microfilaments, dynein, myosin, and the actin cortex (Ouyang et al., 2013; Kirkcaldie and Collins, 2016; Tofangchi et al., 2016). For example, neurofilaments and microfilaments contribute to the axon's elasticity and provide additional mechanical support (Ouyang et al., 2013; Kirkcaldie and Collins, 2016). The actin cortex generates an overall compressive force around the axon that counteracts axonal tension and will likely affect the development of damage (Fan et al., 2017; García-Grajales et al., 2017). The extracellular matrix and the myelin sheaths around the axon provide additional stability and mechanical support (Goriely et al., 2015b; Weickenmeier et al., 2016). Clearly, further experimental and computational research is needed to qualify and quantify the effects of these structural elements on axonal damage. Second, although the Bell model is widely used for a variety of chemical bonds (Evans and Calderwood, 2007), it is not specific to the tau-microtubule complex. The tau-microtubule interaction is largely unknown and an active field of research (Kadavath et al., 2015; Li et al., 2015; Vemu et al., 2016). An improved understanding of the tau-microtubule binding mechanisms and tau-tau interactions can directly feed into our model and will help improving our model predictions. Third, we assume an S-shaped damage evolution as function of axonal stretch that provides a good representation of the numerical simulation results. However, our current S-shaped curve does not explicitly enforce that a zero damage condition at no loading. In principle, we could use any other damage evolution law to model the stretch- and stretch rate-dependent evolution of damage. Fourth, our method of quantifying axon *damage* is based on the excessive detachment of crosslinks caused by an externally applied stretch. Within our computational model, however, this axonal damage may recover when the axon is unloaded or when the stretch is held constant and crosslinks reattach to the microtubules. This recovery is non-standard in continuum damage mechanics. Future research should investigate this issue in more detail to improve on the dynamic mechanism assigned to our crosslinks. For example, crosslinks that detach due to an excessive force may not be able or allowed to reattach again, which could be consistent with the experimentally observed increase in tau protein concentration upon axonal damage.

## 5. Conclusion

The interplay of protein dynamics and physical forces is critical to understand the underlying mechanisms of axonal degradation and brain damage. Here we provides a systematic strategy to relate the discrete dynamic behavior of tau crosslinks on the protein level to the progressive structural degradation on the cellular level to a continuum damage model on the tissue level. Consistent with the definition in nonlinear mechanics, we interpret damage as the gradual stiffness degradation that emerges naturally from a net reduction of crosslinking tau proteins. Motivated by molecular mechanisms, the evolution of damage depends on both the axonal stretch and stretch rate. The only unknown parameter in our model is the characteristic crosslink bond strength, which we vary systematically over two orders of magnitude. Strikingly, for a wide range of stretches, from 1.0 to 1.5, stretch rates, from 0.1 to 10 /s, and bond strengths, from 1 to 100 pN, our model predicts a rather narrow window of critical damage thresholds from 1.01 to 1.30. These values agree well with the experimentally observed axonal damage thresholds reported in the literature. We anticipate that our biophysical model will improve our fundamental understanding of the development and propagation of brain damage across scales and provide useful guidelines to characterize the critical damage level thresholds in response to physical forces.

## Author contributions

RdR performed the simulations. RdR and EK designed the study, analyzed data, and wrote the manuscript.

### Conflict of interest statement

The authors declare that the research was conducted in the absence of any commercial or financial relationships that could be construed as a potential conflict of interest.
